# Tracing the evolving dynamics and research hotspots of spinal cord injury and surgical decompression from 1975 to 2024: a bibliometric analysis

**DOI:** 10.3389/fneur.2024.1442145

**Published:** 2024-08-05

**Authors:** Siqiao Wang, Wei Xu, Jianjie Wang, Xiao Hu, Zhourui Wu, Chen Li, Zhihui Xiao, Bei Ma, Liming Cheng

**Affiliations:** ^1^Division of Spine, Department of Orthopedics, Tongji Hospital Affiliated to Tongji University School of Medicine, Shanghai, China; ^2^Key Laboratory of Spine and Spinal Cord Injury Repair and Regeneration (Tongji University), Ministry of Education, Shanghai, China; ^3^Institute of Spinal and Spinal Cord Injury, Tongji University School of Medicine, Shanghai, China; ^4^Stem Cell Translational Research Center, Tongji Hospital, Tongji University School of Medicine, Shanghai, China

**Keywords:** spinal cord injury, surgical decompression, functional recovery, bibliometric analysis, bibliometrix

## Abstract

**Background:**

Exploration of the benefits and timing of surgical decompression in spinal cord injury (SCI) has been a research hotspot. However, despite the higher volume and increasing emphasis on quality there remains no bibliometric view on SCI and surgical decompression. In this study, we aimed to perform bibliometric analysis to reveal the core countries, affiliations, journals, authors, and developmental trends in SCI and surgical decompression across the past 50 years.

**Methods:**

Articles and reviews were retrieved from web of science core collection between 1975 and 2024. The bibliometrix package in R was used for data analysis and visualizing.

**Results:**

A total of 8,688 documents were investigated, indicating an ascending trend in annual publications. The USA and China played as the leaders in scientific productivity. The University of Toronto led in institutional productions. Core authors, such as Michael G. Fehlings, showed high productivity, and occasional authors showed widespread interests. Core journals like *Spine* and *Spinal Cord* served as beacons in this field. The interaction of core authors and international collaboration accentuated the cross-disciplinary feature of the field. Prominent documents emphasized the clinical significance of early decompression in 24 h post SCI.

**Conclusion:**

Based on comprehensive bibliometric analysis and literature review, we identified the hotspots and future directions of this field: (1) further investigation into the molecular and cellular mechanisms to provide pre-clinical evidence for biological effects of early surgical decompression in SCI animal models; (2) further evaluation and validation of the optimal time window of surgical decompression based on large cohort, considering the inherent heterogeneity of subpopulations in complicated immune responses post SCI; (3) further exploration on the benefits of early decompression on the neurological, functional, and clinical outcomes in acute SCI; (4) evaluation of the optimal surgical methods and related outcomes; (5) applications of artificial intelligence-based technologies in spinal surgical decompression.

## 1 Introduction

Spinal cord injury (SCI) is devastating, which significantly compromises the life quality of affected patients. Approximately 500,000 new SCI cases per year were reported worldwide with young population accounting for the majority of these cases (1). Patients’ symptoms and paralysis resulted from SCI are known as one of the conditions that leads to serious mental and physical impairment. From financial and social points of view, the long-term hospitalization and high cost for therapy imposes a huge burden on patients with SCI and their care givers, which causes great burdens on healthcare systems (2). Hence, it is of necessity to investigate effective treatment strategies for preventing secondary damages and improving functional recovery in SCI patients. SCI treatments have made significant progress in the past years based on the exploration of molecular mechanisms, pathophysiology, neural regeneration, and the improvement of surgery (3, 4). After early operative decompression was proposed in brain injuries (5), early surgical decompression has been widely explored in SCI. Nevertheless, appropriate methods and time window of decompression for SCI remain controversial.

The field of SCI and surgical decompression has produced a variety of significant researches in past decades. These researches were spread over many journals, making it difficult to determine the most impactive sources in the field of SCI and surgical decompression. To date, no research has conducted bibliometric analysis to realize knowledge mining and comprehensively explore the field of SCI and surgical decompression. Hence, we performed a bibliometric analysis to further explore the associated researches, which can reveal significant insights in this dynamic field over the past decades.

Web of Science (WOS) is a commonly used database for technical and scientific literature (6), which help scholars to comprehensively evaluate citation frequency and establish reference co-citation networks for specific research fields. Bibliometrics is a powerful tool for investigating scientific output, developmental trends, and impacts, through evaluating publication characteristics, the geographical distribution feature of research endeavors, critical sources, collaboration networks, and development trends (7). Recently, bibliometric analyses have been performed in multiple clinical fields, including rheumatism, endocrinology, and trauma (7–9). However, the bibliometric views on the research field of SCI and surgical decompression remained a black box. Here, through performing a comprehensive bibliometric analysis, we investigated the research articles and reviews regarding SCI and surgical decompression to determine the knowledge structure, hotspots, and developmental trends in this field.

## 2 Methods

### 2.1 Data source

**Figure 1:**
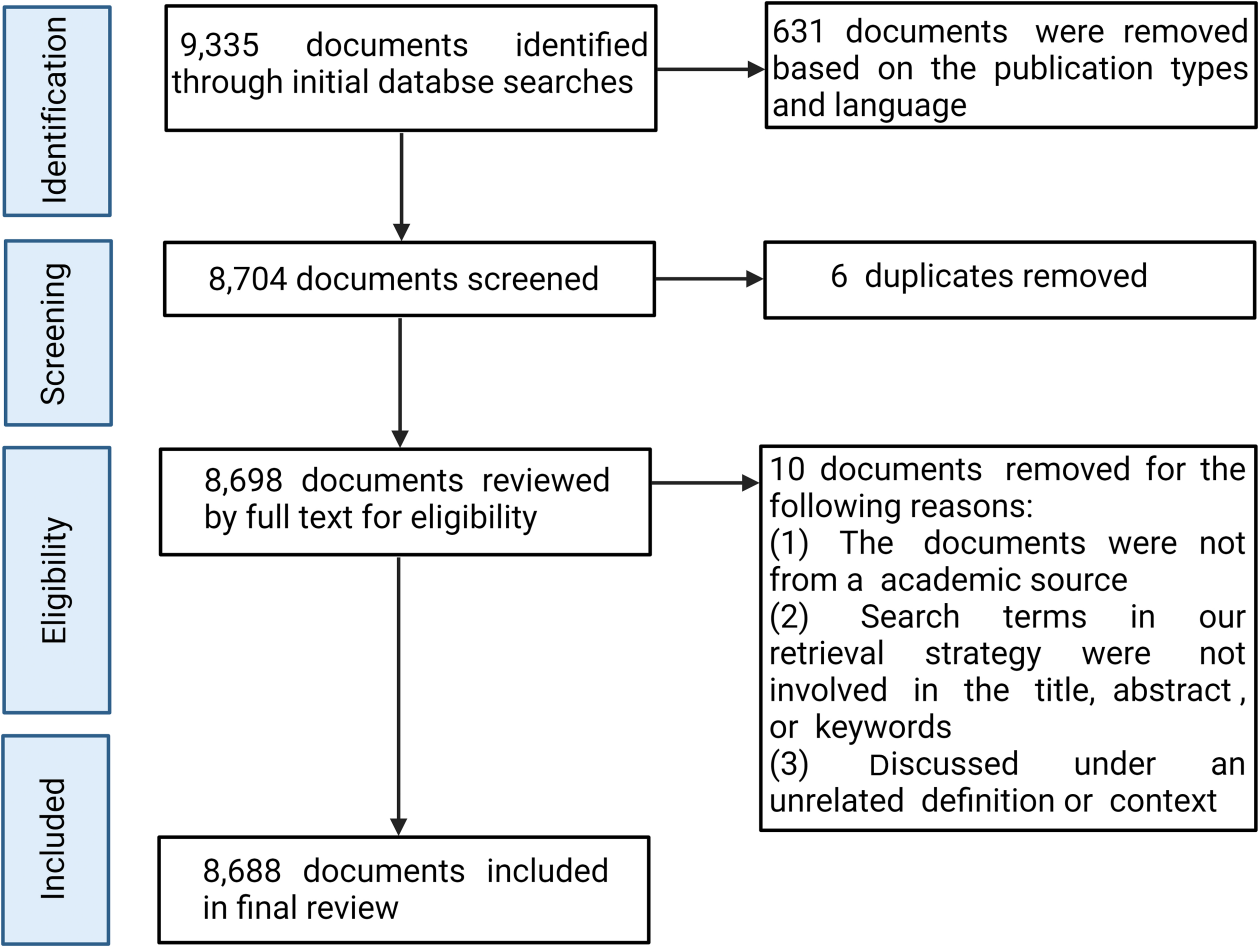
Preferred Reporting Items for Systematic Reviews and Meta-Analyses (PRISMA) flow chart detailing the review process and selection of final sample of publications.

The Web of Science™ (Clarivate™, Philadelphia, PA, United States) was utilized to perform publication retrieval on 14th July 2024. The data sources and inclusion and exclusion criteria were outlined in Figure 1. We utilized Preferred Reporting Items for Systematic Reviews and Meta-Analyses (PRISMA) to document the search processes and results (10).

### 2.2 Screening strategies

The retrieval strategy was designed as follows: (TS = “spinal cord injury” OR TS = “spinal injuries” OR TS = “spinal cord injuries” OR TS = “spinal injury” OR TS = “spinal cord trauma” OR TS = “spinal cord traumas” OR TS = “spinal cord laceration” OR TS = “spinal cord lacerations” OR TS = “post-traumatic myelopathy” OR TS = “traumatic myelopathies” OR TS = “spinal cord contusion” OR TS = “spinal cord contusions” OR TS = “spinal cord transection” OR TS = “central cord injury”) AND (TS = “decompression” OR TS = “surgical” OR TS = “surgery” OR TS = “anterior cervical discectomy and fusion” OR TS = “interbody fusion” OR TS = “arthrodesis” OR TS = “ACDF” OR TS = “endoscopic decompression” OR TS = “discectomy” OR TS = “laminectomy”). Documents published from 1st January 1975 to 14th July 2024 were collected from the WOS Core Collection (WOSCC). Reviews and articles in English were obtained from various publication types.

After excluding documents based on the inclusion criteria of publication types and language, 8,704 documents were extracted. Then, we exported all resulting citations and manually removed duplicates using a bibliographical database manager (EndNote X9). Following duplicate removal, each author reviewed the remaining articles and reviews based on the inclusion criteria independently. Articles and reviews meeting the exclusion criteria were then removed. If any decisions diverged, the research group discussed the results until a consensus was reached.

### 2.3 Content analysis and the base of extraction

We conducted a content analysis according to the title and abstract, which allowed us to categorize and extract the articles and reviews that were in accordance with the research topics, where more than one theme per article could be extracted for further analysis. The above retrieval strategy was used as a guiding framework for our scoping review. Each article was reviewed by at least two authors on separate occasions to increase replicability of our results. The raw data could be seen in Supplementary materials 1, 2. To prevent bias caused by update of WOSCC, data retrieval and collection were performed on 14th July 2024, which were subjected to bibliometric tools for quality evaluation and subsequent analysis.

### 2.4 Data analysis

All retrieved documents were exported in a TXT file format, which were processed using “Bibliometrix” package (version 3.2.1) in R (version 4.3.2, Institute for Statistics and Mathematics, Vienna, Austria; www.r-project.org) to perform comprehensive visualization and knowledge mapping (11). Biblioshiny, a web application for bibliometrix, was used to analyze and visualize the raw data. Annual scientific production was investigated to show the general trend within the field of SCI and surgical decompression. The most influential countries, affiliations, authors, and journals were determined by the scientific productions as well as local/global citations. Further, algorithms including Lotka’s law (12), Bradford’s law (13), and measurement like h-index (14) were utilized to determine the core journals and authors. Lotka’s law defines that the number of authors who make n contributions is approximately 1/n^a^ of those with one contribution, where a is usually almost two which indicates that the number of authors contributing a particular number of papers is inversely proportional to the number of contributed papers (15). The Lotka’s law explains the scientific productivity and the relationship between the authors and their papers through predicting the contribution of an author for a publication. Bradford’s law describes the scatter of citations in a given research field, which can be utilized to determine the most highly cited journals or the core journals in a specific research field (16). The h-index, defined as an author’s h papers with at least h citations, is utilized as a measure of academic influence in bibliometrics (17). Countries’ collaboration of was analyzed to determine the global correlations among different countries. To identify the hot topics, after capturing keywords with high occurrence frequency and the most cited articles, a keyword co-occurrence network was established to show the concrete content of the hotspots. A tree map was constructed to show the most frequent keywords. A thematic map was established to show the different themes through performing a clustering algorithm. The x-axis indicated the centrality (the significance of themes) and the y-axis indicated the density (theme’s development degree). Each bubble referred to a theme cluster and the bubble size indicated their occurrences. Thematic evolutions were investigated by dividing the time span into four different slots (1975–2009, 2010–2014, 2015–2019, and 2020–2024).

## 3 Results

### 3.1 Literature search results

The initial literature search yielded a total of 9,335 documents published between 1st January 1975 and 14th July 2024 from the WOSCC. A total of 8,704 articles and reviews written in English were extracted. After removing 6 duplicates and 10 documents failing to meet the inclusion and exclusion criteria, we obtained 8,688 articles and reviews in the final list of publications used for bibliometric analysis.

### 3.2 Analysis of annual publications

**Figure 2:**
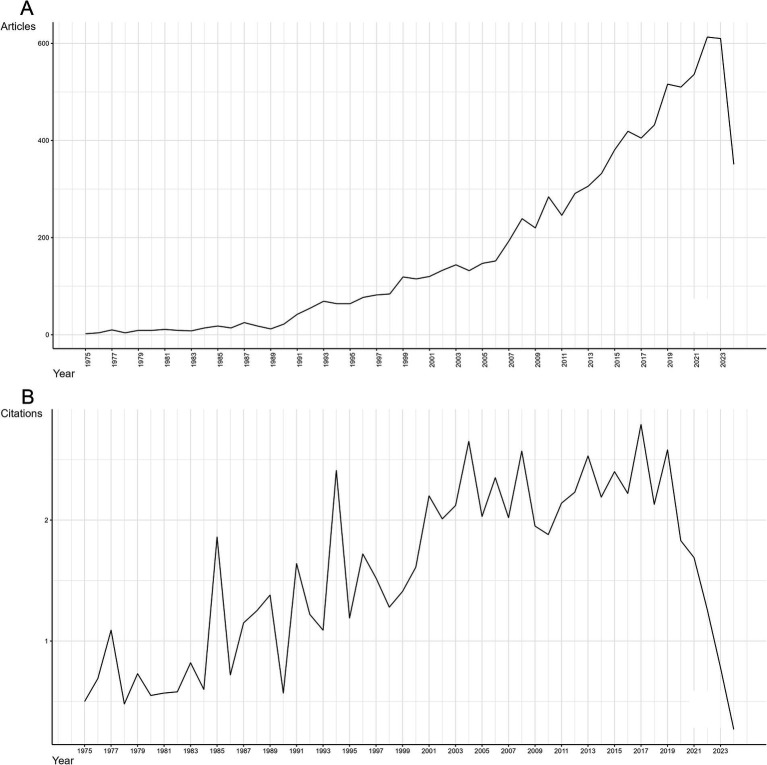
Analysis of annual scientific production and annual citation of documents on spinal cord injury (SCI) and surgical decompression. **(A)** The manifestly explosive growth of annual production started from 1991, and the high peak indicated scientific breakthroughs. **(B)** The trend of annual citations per year in the field of SCI and surgical decompression. The high peak implicated that significant research outcomes were gained in this field.

Data retrieval strategies and analysis processes were illustrated in Figure 1. From 1st January 1975 to 14th July 2024, 8,688 documents were retrieved from WOSCC. A total of 8,688 documents that met the inclusion criteria including 7,171 (82.5%) research articles and 1,517 (17.5%) reviews were obtained for the subsequent analysis. The data retrieval date (14th July 2024) accounted for the steep decline in 2024 (Figure 2A). Before 1991, only a limited number of researches were published. Then, from 1991 to 2005, an intermittent and slow increase in annual publications were identified. The most significant increases happened in 2010s, with over 200 papers published per year in this field. Generally, the annual growth rate of publication number was approximately 11.2%, and the average age of documents was about 10.8 years. The growing trend indicated that the field of SCI and surgical decompression was becoming more and more attractive. The trend of annual citations per year in the field of SCI and surgical decompression was shown in Figure 2B. The high peak of the years distribution of citation appeared in the period between 2004 and 2019, implicating that significant research outcomes were gained in this field. The average citations per document was approximately 22.8. However, the overall citation frequency has rapidly declined in the past 5 years.

### 3.3 Analysis of countries, affiliations, and authors

**Figure 3:**
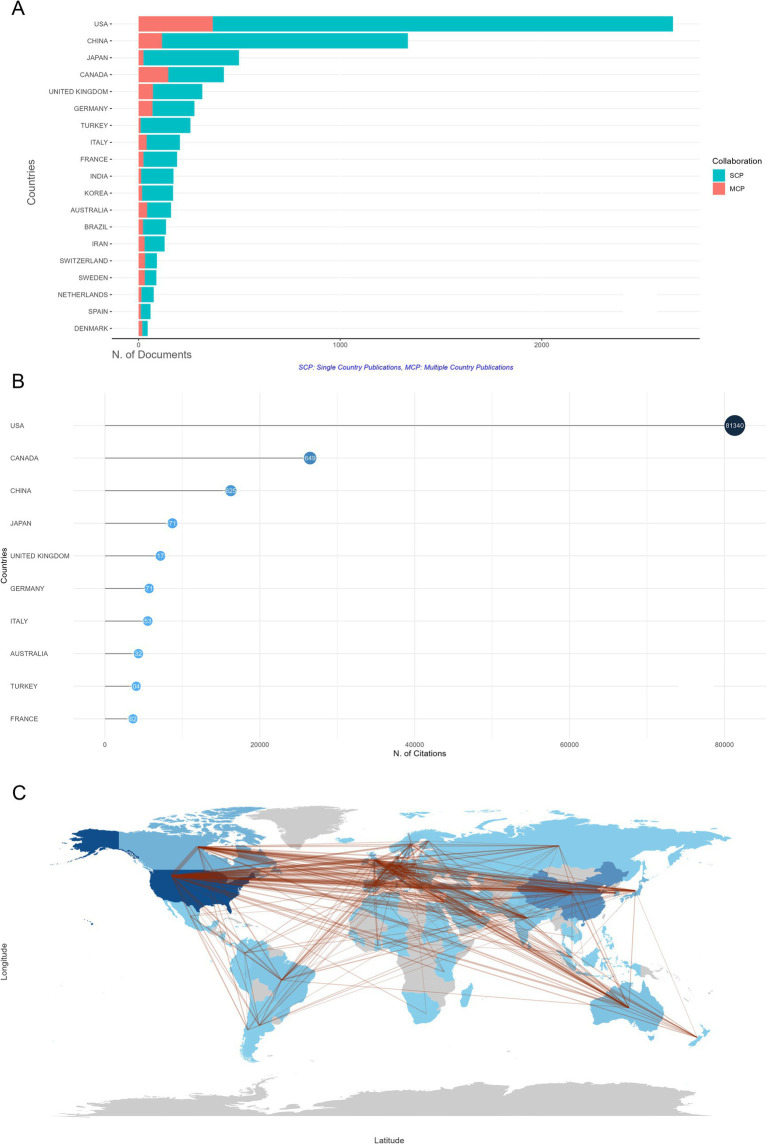
Analysis of countries in the field of spinal cord injury (SCI) and surgical decompression. **(A)** Countries’ scientific production and collaboration histogram based on the nationality statistics of the corresponding authors in SCI and surgical decompression studies. SCP, single country publications. MCP, multiple country publications. **(B)** Top 10 most cited countries in SCI and surgical decompression researches. **(C)** Country collaboration map in SCI and surgical decompression researches.

A total of 88 countries contributed to the field of SCI and surgical decompression. Ranked by accumulated publication number, the top 3 most prolific countries were the USA (*n* = 2,651 30.5%), China (*n* = 1,336, 15.4%), and Japan (*n* = 498, 5.7%). Collaboration strength among different countries can be revealed by the single-country publication (SCP) and multiple-country publication (MCP) rates. Countries showing the highest MCP ratio included the USA, China, Canada, the United Kingdom (UK), and Germany; other countries, such as Japan and Turkey, mainly provided the domestically published articles. Nevertheless, with high number of productions, the overall citation number of articles from China (*n* = 16,257) was considerably less than the USA (*n* = 81,340), which ranked the third among the contributing countries (Figure 3B), indicating the huge influence of the USA within the research field. Besides, Canada ranked the second with 26,492 citations. Country collaboration analysis showed that, China and the USA had the most interactions with others (Figure 3C). There were 819 collaborations across the contributing countries, 74 of which were from the USA to others. In the collaboration map, the connection between China and the USA was the most extensive, highlighting the interactions between these two countries for SCI and surgical decompression-related researches. Generally, the lines between countries were scattered and sparse, indicating the multiple country publications were less than the single country publications.

**Figure 4:**
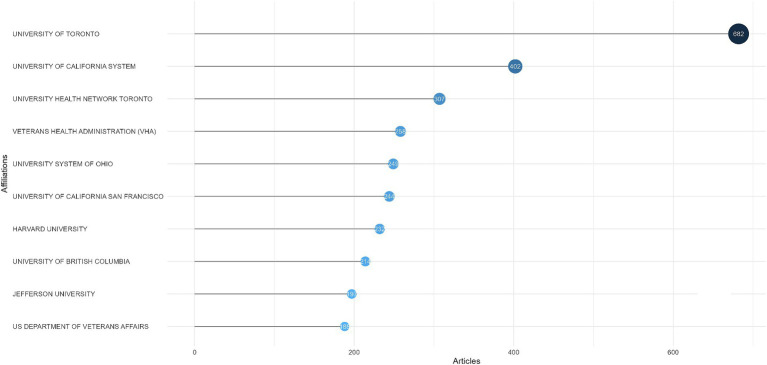
Top 10 most productive institutions in spinal cord injury (SCI) and surgical decompression researches.

Currently, a total of 4,527 institutions have participated in the field of SCI and surgical decompression, and the top 10 most prolific institutions were identified (Figure 4). It indicated that University of Toronto ranked the first, with 682 publications, followed by University of California System (*n* = 402), University Health Network Toronto (*n* = 307), Veterans Health Administration (*n* = 258), and University System of Ohio (*n* = 249). These findings were consistent with the results discussed above, which showed the USA and Canada were the pioneers in the field of SCI and surgical decompression.

### 3.4 Analysis of journals

**Figure 5:**
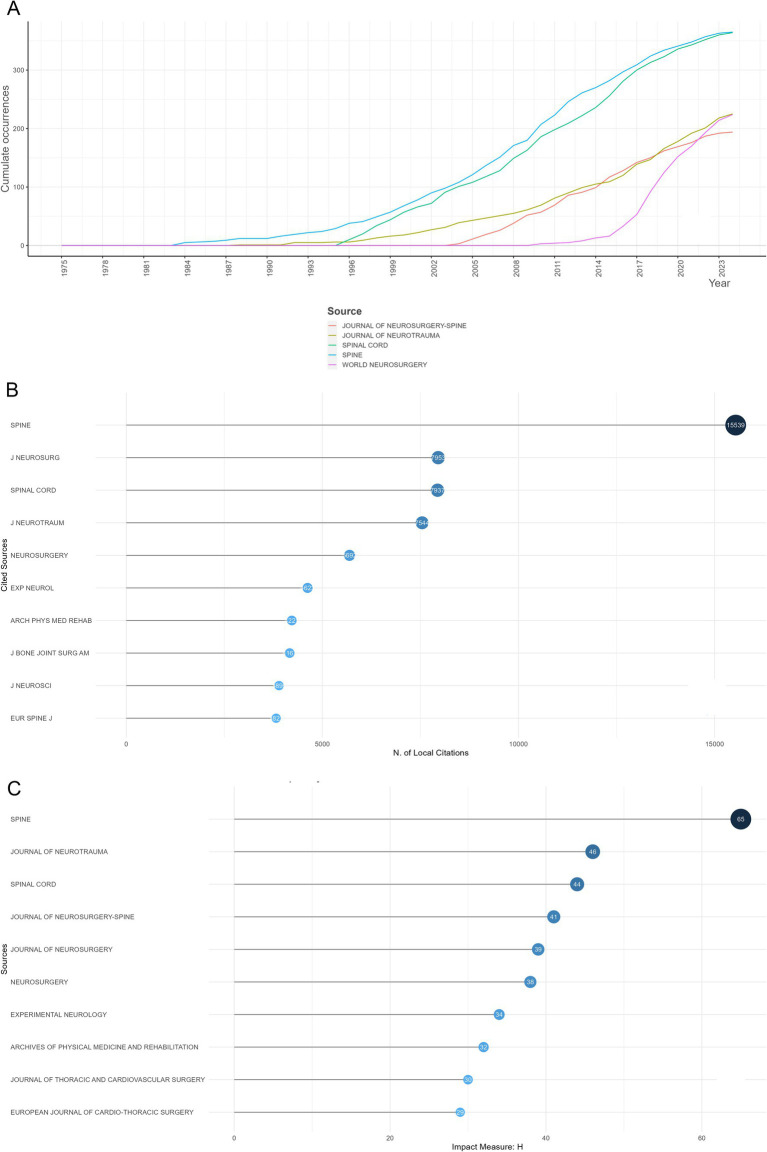
Analysis of journals in the field of spinal cord injury (SCI) and surgical decompression. **(A)** The publications’ growth of top five productive journals in the field of SCI and surgical decompression. **(B)** Top 10 most local cited journals in the field of SCI and surgical decompression. **(C)** Most local impactive journals measured by h-index in the field of SCI and surgical decompression.

Publications and citations were utilized for evaluating the overall influence of 1,545 journals in the field of SCI and surgical decompression. The top 10 most productive journals published a total of 2,012 documents from 1975 to 2024, accounting for about 23.2% of all the documents in this field (Supplementary Figure S2A). According to the Bradford’s law, the top 23 most prolific journals were defined as the core sources in the research field (Supplementary Figure S2B). Specifically, *Spine* (*n* = 365) was the most productive journal, followed by *Spinal Cord* (*n* = 364), *Journal of Neurotrauma* (*n* = 225), *World Neurosurgery* (*n* = 224), and *Journal of Neurosurgery-Spine* (*n* = 194). Among the journals, *Spine* was the earliest one (year 1984, *n* = 5) to publish articles in the field of SCI and surgical decompression, which continued to rise and remained at the top. However, *World Neurosurgery* was the latest one, with quick growth in publication number (Figure 5A).

The number of local citations was evaluated based on the reference list to evaluate the local impact of journals, whereas global citations covered a broad scope of research fields. In terms of local citations, *Spine* ranked the top of the list with 15,539 citations, followed by *Journal of Neurosurgery* (*n* = 7,953) and *Spinal Cord* (*n* = 7,937), indicating these journals provided a variety of superior quality research (Figure 5B). When h-index was calculated to evaluate the local impact of journals, we identified that *Spine* (h-index = 65), *Journal of Neurotrauma* (h-index = 46), and *Spinal Cord* (h-index = 44) were the top three most impactive sources (Figure 5C).

### 3.5 Analysis of authors

**Figure 6:**
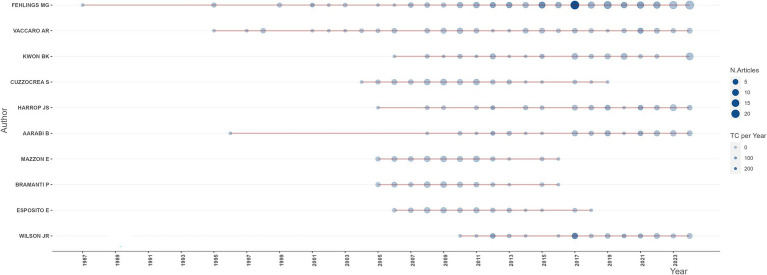
Production of the top 10 productive authors over time in the field of spinal cord injury (SCI) and surgical decompression.

A total of 32,260 authors have published relevant articles on SCI and surgical decompression since 1975, making efforts to facilitate the development of the field. Lotka’s law showed correlations between authors and related publications. In general, it showed the phenomenon that a small proportion of authors provided most of the related articles. Herein, the Lotka’s law was used to evaluate the publications in the field of SCI and surgical decompression, as the majority of the relevant documents in this field were published by a rather small population of authors, and about 80% of authors published only one article related to this research field (Supplementary Figure S3A). The top 10 most productive authors were illustrated in Figure 6, among whom Fehlings MG topped the rankings with a total publication number of 202, who has retained an active role until today. Although starting late, Kwon BK, Harrop JS, and Wilson JR continued to be active till now. Additionally, Fehlings MG was the most local cited authors (*n* = 2,590), followed by Aarabi B (*n* = 989; Supplementary Figure S3B). According to the h-index, the top 10 most impactive authors were illustrated in Supplementary Figure S3C. Further, Fehlings MG and Vaccaro AR were the two most impactive authors in this field with the highest h-index.

### 3.6 Analysis of documents and references

**Figure 7:**
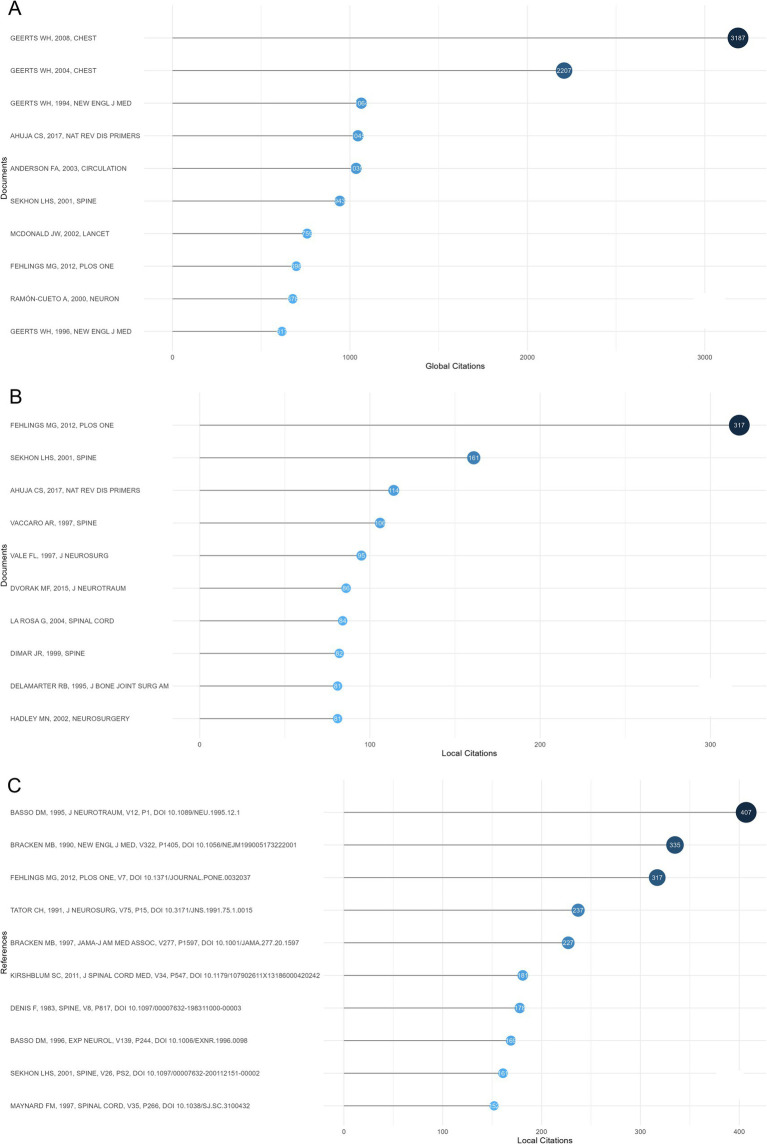
Analysis of documents and references in articles regarding spinal cord injury (SCI) and surgical decompression. **(A)** The top 10 most globally cited documents concerning SCI and surgical decompression. The size and darkness of the nodes are in proportion to the number of global citations of each document. **(B)** The top 10 most locally cited documents concerning SCI and surgical decompression. The size and darkness of the nodes are in proportion to the number of local citations of each document. **(C)** The top 10 most locally cited references concerning SCI and surgical decompression. The size and darkness of the nodes are in proportion to the number of local citations of each document.

Most cited documents and references served as the future directions for investigating SCI and surgical decompression. Globally cited documents were the articles that were cited in all research fields, whereas locally cited documents were only cited in the field of SCI and surgical decompression. Hence, high number of globally cited documents may represent the overall impact, whereas high number of locally cited documents indicated their influence in the field of SCI and surgical decompression. Figures 7A,B summarized the top 10 most globally cited documents as well as the top 10 most locally cited documents in the field of SCI and surgical decompression, respectively. Specifically, the highest globally cited document, “Prevention of venous thromboembolism,” by Geerts WH in 2008 (3,187 global citations) (18), discussed the prevention of venous thromboembolism (VTE). They recommended all major trauma and all SCI patients receive thromboprophylaxis (Grade 1A). In patients admitted to hospital with acute SCI, they recommended thromboprophylaxis with a low-molecular-weight heparin (LMWH), low-dose unfractionated heparin (LDUH), or fondaparinux (each Grade 1A). Furthermore, they recommended that, on admission to the intensive care unit (ICU), all SCI patients be assessed for their VTE risk, and that most of them receive thromboprophylaxis (Grade 1A).

The highest locally cited document, “Early versus Delayed Decompression for Traumatic Cervical Spinal Cord Injury: Results of the Surgical Timing in Acute Spinal Cord Injury Study (STASCIS)” (19), by Fehlings MG, published in 2012 (317 local citations), evaluated the relative effectiveness of early (<24 h after injury) vs. late (≥24 h after injury) surgical decompression post traumatic cervical spinal cord injury (CSCI). They identified that, the odds of at least a 2 grade AIS improvement were 2.8 times higher than those who underwent early surgical decompression as compared with those who underwent late decompressive surgery (OR = 2.83, 95% CI: 1.10,7.28) based on the multivariate analysis. They concluded that, surgical decompression prior to 24 h post SCI could be safely performed, which was related to improved neurologic outcomes.

The top 10 most locally cited references were shown in Figure 7C, “A sensitive and reliable locomotor rating-scale for open-field testing in rats” (20), by Basso DM, published in 1995 (407 local citations), developed an expanded and unambiguous locomotor rating scale for standardizing locomotor outcome measures among laboratories, which can clearly distinguish behavioral outcomes after different injuries and predict anatomical alterations in the injured sites.

### 3.7 Analysis of keywords

**Figure 8:**
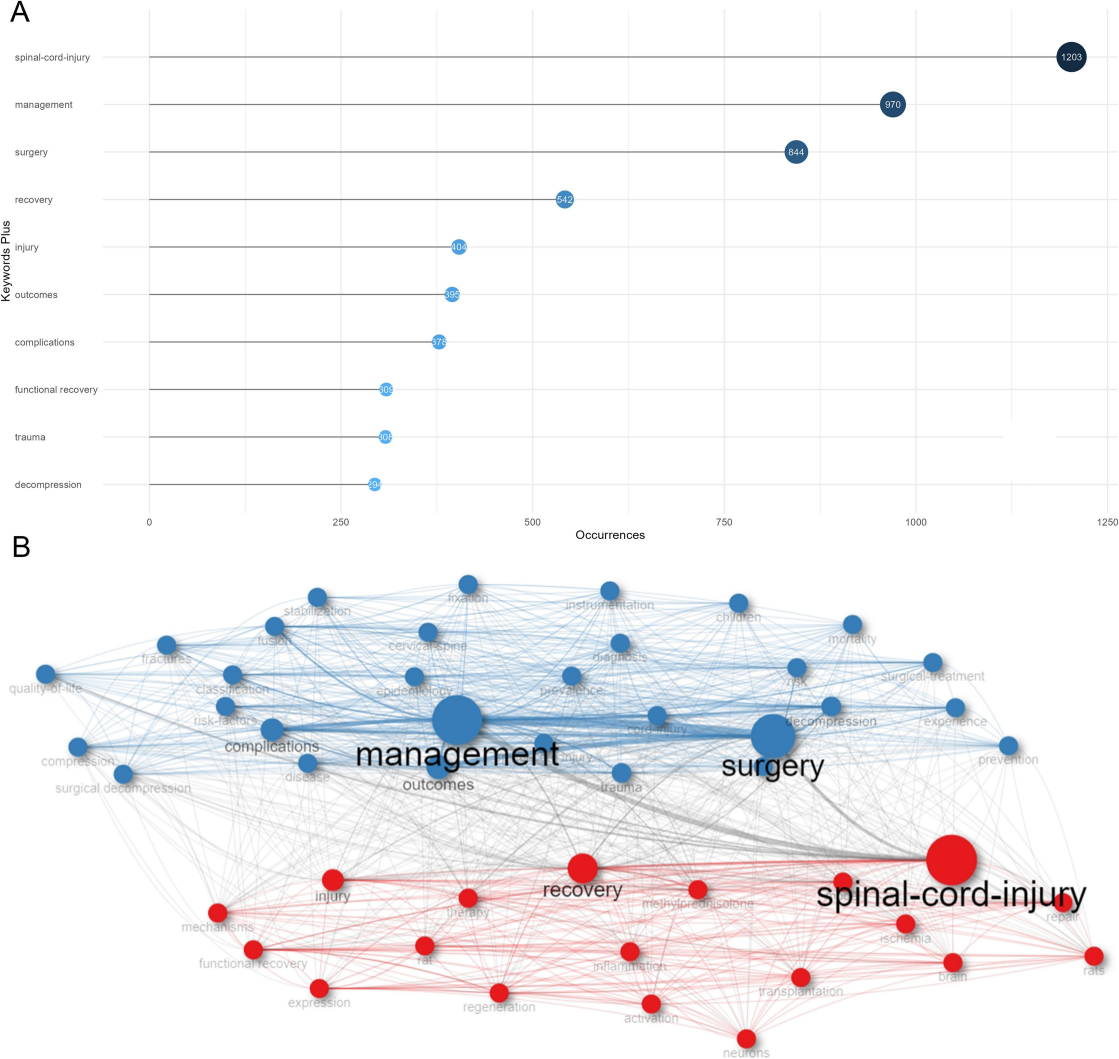
Analysis of keywords in the field of spinal cord injury (SCI) and surgical decompression. **(A)** The top 10 most frequent key words selected from KeyWords Plus. The size and darkness of the nodes are in proportion to the number of occurrences of each keyword. **(B)** Co-occurrence analysis of key words. Keywords were divided into two clusters labeled with different colors (blue, red).

Key words constantly appeared in internalized documents represented the hotspots in a specific research field. As a useful algorithm that is unique to Clarivate databases, KeyWords plus enhances the power of reference searching By searching across disciplines for all the documents that had cited references in common. Based on KeyWords plus analysis (Figure 8A), we identified the top 10 most frequent keywords, including “spinal-cord-injury” (1,203 occurrences), “management” (970 occurrences), “surgery” (844 occurrences), “recovery” (542 occurrences), “injury” (404 occurrences), “outcomes” (395 occurrences), “complications” (378 occurrences), “functional recovery” (309 occurrences), “trauma” (308 occurrences), and “decompression” (294 occurrences), far exceeding other keywords, indicating that this research field concentrated on developing clinically efficient management strategies and surgical methods to improve neurological outcomes, promote functional recovery, and reduce short-term and long-term complications after SCI. The high frequency of appearance of these keywords was further demonstrated and visualized by a keyword tree and word cloud, respectively (Supplementary Figures S4A,B).

A keyword co-occurrence network was constructed to show the internal and external link between the most relevant keywords which occurred more than 10 times in this field (Figure 8A). Link lines indicated the two keywords appeared in one or more articles at the same time. These keywords were grouped into two distinct sub-clusters representing two major themes. Cluster 1 (red) where “spinal-cord-injury,” “recovery,” “injury,” “functional recovery,” “expression,” “methylprednisolone,” “repair,” “model,” “regeneration,” and “rat” were the largest nodes. It chiefly explained the critical role of methylprednisolone in SCI therapy which provided novel therapeutic efficacy and availability in SCI patients and SCI animal models. Cluster 2 (blue) was represented by “management,” “surgery,” “outcomes,” “complications,” “trauma,” “decompression,” “fusion,” “risk,” “risk-factors,” “surgical-treatment,” and “classification,” which concentrated on the risk factors or risk classification models of different clinical outcomes and complications of SCI patients after surgery.

### 3.8 Analysis of emerging trends

**Figure 9:**
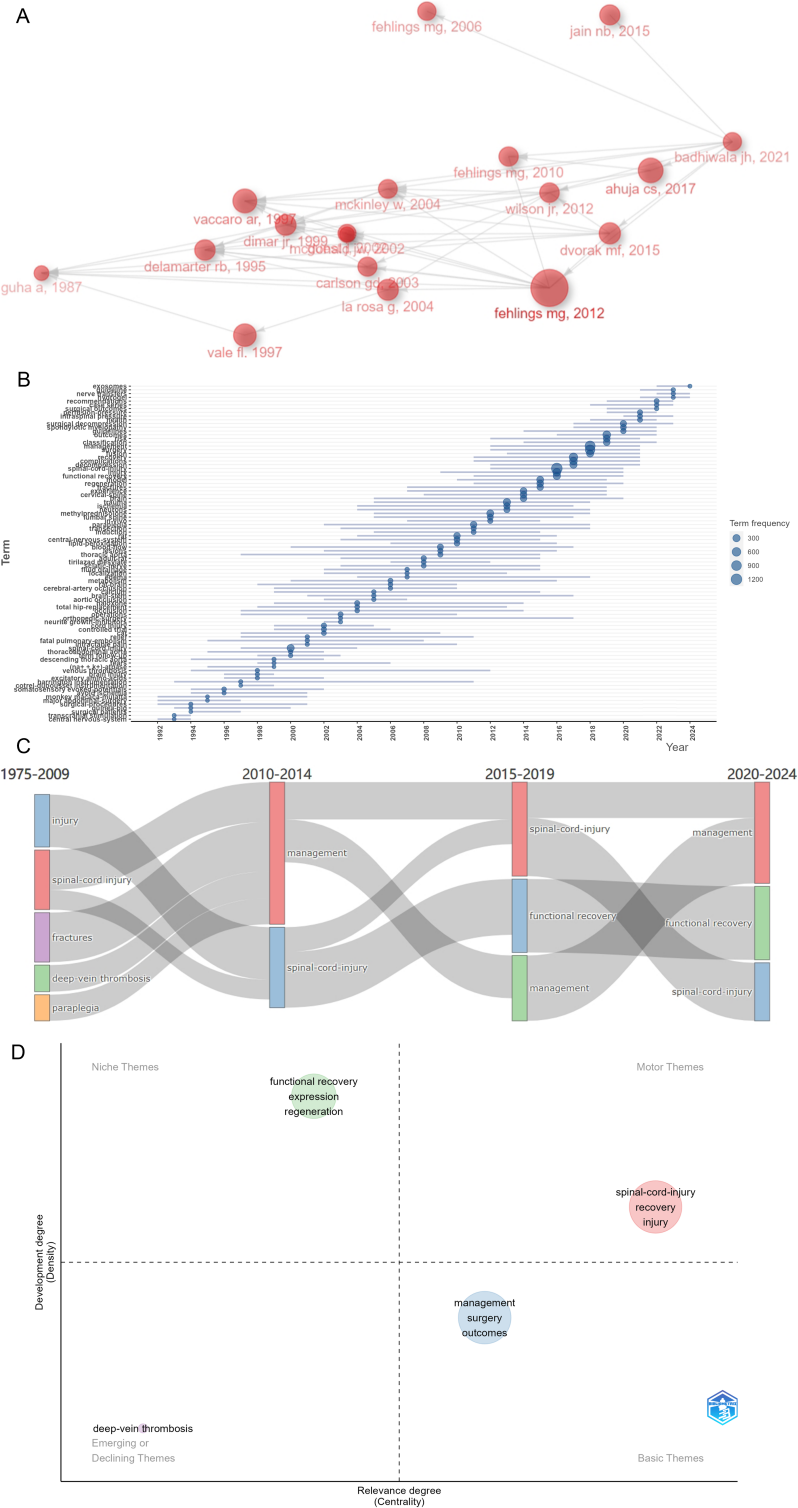
Investigating research status of various hot topics on spinal cord injury (SCI) and surgical decompression researches, sketching historical trajectories and revealing research frontiers. **(A)** Historical direct citation network of key documents in SCI and surgical decompression researches. **(B)** Trend Topics Analysis with the 3 keywords per year which occurred at least 10 times in the past decades. **(C)** Sankey plot of the most frequent key words in different time period (1975–2009, 2010–2014, 2015–2019, and 2020–2024). **(D)** Thematic map for SCI and surgical decompression researches. The horizontal coordinate referred to the relevance degree (centrality), and the vertical coordinate represented the development degree (density). Motor themes in the first quadrant represented core themes with high centrality and maturity, niche themes in the second quadrant represented isolated themes with increased maturity, the third quadrant represented emerging or declining themes with low centrality and high maturity, and basic themes in the fourth quadrant indicated popular themes with low maturity.

Based on the historical direct citation network, connections across most of the highly cited documents were unveiled (Figure 9A). The link lines between documents indicated that the earlier research was cited by the latter one, where “Early versus Delayed Decompression for Traumatic Cervical Spinal Cord Injury: Results of the Surgical Timing in Acute Spinal Cord Injury Study (STASCIS)” (19) was the past and had a pivotal role in the citation network.

To identify the future trend topics in the field of SCI and decompression, several popular keywords in the past three decades were obtained for further analysis. To construct the trend topics map, the minimum keyword frequency was set to 10 and the number of keywords per year was set to three. Finally, 90 trend topics were identified, and the frequency and popular periods of these research topics were visualized (Figure 9B). Specifically, several trend topics, such as “exosomes,” “guideline,” “nerve transfers,” “hydrogel,” “recommendations,” “case series,” “surgical outcomes,” “perfusion-pressure,” “intraspinal pressure,” “health,” and “spondylotic myelopathy” have gained considerable traction over the past 5 years. Dynamic changes within the thematic fields across years (time-period 1975–2009, 2010–2014, 2015–2019, and 2020–2024) were displayed in Figure 9C. Functional recovery is an eternal theme in the field of SCI and surgical decompression. Over time, increasing interests have been attracted by more clinically efficient management strategies of SCI patients who underwent surgical decompression.

### 3.9 Conceptual analysis

In addition to the dynamics of keywords, the possible relationships across themes with high frequencies also had great significance to better understand the hot topics covered by the research field of SCI and surgical decompression and evolution trends over time. A two-dimensional matrix was constructed to visualize the status of the three major themes in the field of SCI and surgical decompression (Figure 9D). The X axis was labeled with “centrality,” representing the relevance degree of the research themes; the Y axis was labeled with “density,” indicating the development degree of the research themes. Further, the bubbles were labeled with the most important keywords with the highest occurrences. Generally, four major research themes were distributed in the four quadrants. The green cluster with high development degree and low centrality was described as niche themes, representing that unique gene expression and neural regeneration to promote the functional recovery of SCI patients were highly developed but less associated with the field. The red cluster with high development degree and high centrality was defined as the motor themes the thematic map. Moreover, the blue cluster was located in the quadrant 4, which represented the basic themes. Additionally, deep-vein thrombosis served as the emerging or declining themes, exhibiting a decreasing trend in this research field.

## 4 Discussion

### 4.1 General information

Herein, we explored the development trends and hotspots over the past 5 decades and investigated the research frontiers within the field of SCI and surgical decompression in recent years. This bibliometric analysis provided a comprehensive outlook on the associated scientific publications, which shed light on the current research status and identified possible avenues for further investigation. Our results aligned with existing publications, showing an increasing scientific output, multinational collaborations, and emerging thematic alterations within this field (3, 21). Over time, the increased number of publications on SCI and surgical decompression indicated the growing recognition of the critical role that early surgical decompression plays in SCI treatments (22). Surgical decompression for acute SCI aims to reduce secondary ischemia and hypoxia through relieving mechanical pressure. A meta-analysis based on 21 pre-clinical researches showed spinal cord decompression improved the neurological outcomes by 35%, and compressive pressure and duration were critical factors affecting the clinical outcomes (23). Further, previous experimental evidence supported that persistent compression may be a reversible form of secondary injuries (24). Despite its widespread use in acute SCI in North America, the role of surgical decompression in improving neurologic function remained unclear due to the absence of well-designed randomized controlled trials (RCTs). Moreover, the optimal therapeutic window, during which operative decompression could mitigate the secondary SCI, also remained controversial. The logistical and practical challenges associated with early surgical decompression of acute SCI remained important problems.

The wide range of journals publishing on this field, with *Spine*, *Spinal Cord*, and *Journal of Neurosurgery* emerging as the top sources. It indicated that experts from multiple disciplines were actively engaged in this field (25, 26). Such collaboration promoted the depth and breadth of researches on the topic. The analysis of authors revealed the contributions of core authors and occasional authors to the publications related to SCI and surgical decompression. The presence of core authors, such as Michael G. Fehlings and Alexander R Vaccaro, signified their expertise and high productivity within the field. Moreover, the large number of occasional authors suggested a wide engagement of scholars, which emphasized the growing interests in investigating the role of surgical decompression in SCI. Evaluating affiliations provided important insights in the institutional landscape of SCI and surgical decompression research. The prominence of the University of Toronto as the leading affiliation in aspects of publication number indicated a concentrated effort in the field of SCI and surgical decompression. Further, the involvement of institutions from Canada and the USA across the most productive contributors underscored the global collaboration of research on SCI and surgical decompression. The multinational collaboration fostered the exchange of knowledge and promoted the pooling of resources.

The analysis of country scientific output highlighted the USA, China, and Japan as the leading countries in this field. Besides, other countries have also played an important part in the scientific collaboration network, indicating a global effort to overcome the challenges from surgical decompression in SCI. Several experts proposed that, though the recommendations for early surgical decompression in specific patients with acute SCI are well recognized, there is uncertainty about the timing of spinal cord decompression in SCI managements (21). The most locally cited documents provided critical insights in researches that have shaped the research field of SCI and surgical decompression (2, 19, 21, 27–29). The prominent literature entitled “Early versus Delayed Decompression for Traumatic Cervical Spinal Cord Injury: Results of the Surgical Timing in Acute Spinal Cord Injury Study (STASCIS)” represented the most cited and impactive research (19). Fehlings et al. (19) conducted an international, multicenter, prospective cohort study in adults with CSCI aged between 16 and 80. Their results indicated that surgical decompression within 24 h post SCI could be safely conducted and is related to better clinical outcomes (19). Vaccaro et al. (28) performed a prospective analysis to evaluate neurologic outcomes after early vs. late decompressive surgery for CSCI. It revealed no significant neurologic benefits when surgical decompression after CSCI is performed less than 72 h post injury as compared with waiting longer than 5 days. Vale et al. (29) used combined medical and surgical treatments after acute SCI, which demonstrated that early and aggressive medical managements (blood pressure augmentation and volume resuscitation) of patients with SCI can effectively promote the neurological recovery. Fehlings et al. (30) reported that the role of early surgical decompression in SCI patients was only supported by Class III and limited Class II evidence, which can be considered as a practice option only. More prospective and controlled trials are required to investigate the timing and role of early surgical decompression in patients with acute SCI. Furlan et al. (21) reviewed the pre-clinical and clinical evidence regarding the possible influence of timing of decompressive surgery on clinical outcomes post traumatic SCI. There is evidence to demonstrate that early surgical decompression is feasible and safe and that it can optimize neurological outcomes and decrease health care costs (21). Early surgical decompression should be considered in all SCI patients from 8 to 24 h after acute SCI. Ahuja CS et al. also concluded that early surgical decompression requires to be quickly performed after acute SCI (2).

Keyword analysis and thematic clustering provided intriguing insights in the current state of thematic field. The clusters related to spinal cord injury, management, surgery, complications, recovery reflected the multifaceted characteristics of surgical decompression and its influence on SCI functional recovery (3, 31, 32). These clusters highlighted the requirement to investigate the fundamentals, clinical efficiency, post-operative complications of spinal cord decompression. While this study provided critical insights, some research gaps and future directions emerged from this bibliometric analysis. First, there is an urgent need for further exploration on the molecular and cellular mechanisms to provide pre-clinical evidence for biological benefits of early surgical decompression in SCI animal models. It necessitated performing well-designed mechanistic researches, investigating the interplays between surgical decompression and neurological outcomes, and revealing the underlying signaling pathways. Second, there is still much to be explored in terms of the optimal time window for decompression on basis of the current clinical evidence, considering the inherent heterogeneity in complicated inflammatory responses post SCI. Third, further research is warranted to explore the benefits of early surgical decompression on the neurological, functional, and clinical outcomes in SCI.

### 4.2 The role and timing of early surgical decompression in SCI

Unfortunately, there is no high evidence level analysis investigating the safety and efficacy of early surgical decompression in subpopulations with distinct clinical features (33). Nevertheless, in several analyses, the usefulness of early decompression was supported at the suggestion level (34, 35). In subpopulations according to age, no research compared the efficacy and safety of early surgical decompression and late surgical decompression between younger and elder individuals. Recently, there is an analysis evaluating the clinical outcomes of early decompression between younger and elder patients with acute SCI. Lau et al. (36) reported that, the mortality rate and complication rate of elder patients with acute SCI (≥70 years) were 11 times and 2 times higher than younger patients (<70 years). Nevertheless, there was no statistical difference in discharge American Spinal Injury Association impairment scale (AIS) grade and AIS grade alteration between these two patient groups. In subpopulations according to injury degree, SCI patients with AIS grade B, C, and D at C2-L2 level prior to surgical decompression showed a better motor functional recovery with early decompression (≤24 h) than those with late surgical decompression (>24 h). In SCI patients with AIS grade A, there was no significant benefit of early surgical decompression on motor functional recovery. In SCI patients with AIS grade A or B, early surgical decompression contributed to shorter hospital stay (37). Moreover, as AIS grade altered from A to D at admission, when surgical decompression was conducted in 12 h, the conversion rate from AIS grade at admission to better discharge AIS grade was significantly higher (38). Further, ultra-early surgical decompression (≤12 h) exhibited better improvement in the AIS grade than surgical decompression post 12 h. Particularly, in SCI patients with AIS grade A, if ultra-early surgical decompression was conducted, the AIS grade may improve to stage 1 or 2 post operation (38). Though only for CSCI, other researches showed different time windows of surgical decompression (ultra-early, ≤12 h; early, 12–24 h; and late, >24 h) did not influence conversion of AIS grade (38). Further, in subpopulations according to the preoperative use of steroid pulse, early surgical decompression (≤24 h) in SCI patients with preoperative steroid pulse exhibited better improvement in light touch score, pin prick score, and motor functional recovery than late surgical decompression (>24 h). However, there was no notable difference in the improvement of total motor score between early and late surgical decompression in SCI patients who did not use steroid pulse prior to operation (38).

The optimal time window of surgical decompression post SCI still remained controversial (39). It was challenging to standardize the time window because of inherent heterogeneity in the exact definition of “early decompression” for previous studies, and there were limited studies at high level of recommendation. Importantly, most studies set the time window of definition of “early” as 24 h, which was considered to be the most appropriate method to define “early” (38). Moreover, in 2012, the Surgical Timing in Acute Spinal Cord Injury Study (STASCIS) defined “early” as the first 24 h post SCI, and showed surgical decompression in 24 h post injury was related to better neurologic outcomes, exhibiting more than grade 2 AIS improvement in 6 months follow up (19, 38). Subsequently, various researches showed the beneficial effects of early surgical decompression (<24 h) in SCI patients (21, 38). Nevertheless, despite compelling evidence, timing of decompression for patients with acute SCI was still uncertain. Particularly, in those with central cord syndrome without instability, since spontaneous improvement might happen, surgical decompression of the injured spinal cord might cause worsening neurologic function. Arbeitsgemeinschaft für Osteosynthesefragen spine (AOSpine) evaluated the safety and efficacy of early decompression (≤24 h) and late decompression (>24 h) in SCI patients (35). They reported early surgical decompression should be considered as an important therapeutic strategy in adult SCI patients with traumatic central cord syndrome, and early surgical decompression should be performed in adult patients with acute SCI at any level.

Grossman et al. (40) showed if patients visited the emergency center immediately post SCI, most arrived in 4 h, and that 8 h to spinal cord decompression was critical for achieving better clinical outcomes. In this context, an 8-h time threshold was further proposed. Recent RCTs showed that (41), surgical decompression in 8 h in patients with SCI exhibited more neurological improvement than more than 8 h. Additionally, a previous analysis compared the surgical decompression in 8 h vs. 8 to 24 h, which indicated that neurobehavior outcomes were better in surgical decompression in 8 h than 8 to 24 h post SCI (42). Recently, various researches supported the favorable effect of surgical decompression in 8 h post SCI (43–45). Nevertheless, in several researches, “in 8 h post SCI” was defined as the time taken post arriving in the emergency center. Hence, it was hard to compare the conclusions draw from the previous studies directly. Furthermore, within the narrow time window of 8 h post injury, loss of just 1 h is of vital importance. In clinical practice, decompression in 8 h post SCI was quite difficult because of transport time to the emergency center and lack of available operation rooms. However, a previous study showed negative views on early surgical decompression. Tanaka et al. (46) divided patients in an early decompression group (≤24 h) and a late compression group (>24 h to 1 week). The intensive care unit (ICU) stay, survival rate, and mortality rate of patients showed no significant difference between these two groups (46).

In summary, “time is spine,” and the timing of surgical decompression is one of the most significant factors in the treatment of acute SCI. CSCI showed significant improvement with early surgical decompression both in the upper extremities and lower extremities. Early surgical decompression also contributes to better motor outcomes in thoracic SCI as compared with late decompression. Numerous multicenter studies demonstrated that, early surgical decompression significantly promotes long-term neurological improvements (37). Patients with acute SCI should receive decompressive surgery in a center from qualified spinal surgeons as early as possible, but at least in 24 h. Recently, the superiority of the neurological outcomes with decompressive surgery in 8 h has been demonstrated.

### 4.3 The impact of different surgical methods on different injury segments concerning clinical outcomes

Traumatic SCI is usually unstable, so surgical stabilization is particularly important (32). The aims of surgical therapies post SCI include the stabilization and reduction of the secondary SCI while achieving the anatomical alignment of spine, in which surgical decompression plays a critical role. The surgical procedure promotes early mobilization and rehabilitation. Based on the lesions of the fracture, surgical decompression could be performed posteriorly, anteriorly, or in combination procedures (47).

#### 4.3.1 Optimal surgical methods for CSCI

Early surgical reduction and decompression is critical for CSCI. It is always recommended to restore stability or fusion of the injured segments (48). Atlas fractures happen less frequently in combination with SCI because of the larger diameter of the spinal canal. Atlas arch fractures with a dislocation of at least 7 mm usually indicate the instability of the spinal injury. It is recommended to perform instrumentation of ventral, direct dorsal fusion or dorsal osteosynthesis from C1 to C3. Further, axis fractures are rarely accompanied by SCI. In unstable axis fractures, dorsal stabilization is indicated. Besides, ventral screw osteosynthesis is an alternative, whereas it may play a subordinate role in SCI. Traumatic spondylolisthesis of the C2 and C3 vertebral bodies, also called “hangman’s fracture,” with an inclination of vertebral bodies of more than 11° or a dislocation of more than 3.5 mm are recommended for surgical stabilization. Anterior discectomy and fusion (ACDF) and posterior fusion using transpedicular dorsal instrumentation has demonstrated satisfactory fusion outcomes (49).

CSCI with disc protrusion requires an anterior approach with early surgical decompression and one-stage fusion utilizing vertebral body replacement or a cage and concomitant ventral plate osteosynthesis (47). A previous study of sub-axial CSCI treated with posterior and anterior stabilization and fusion showed no difference in position, fusion rates, neurological outcomes, or post-operation complications. The approach route with which the respective surgeon is familiar is recommended (50).

The gentle access route from ventral has proven effective in CSCI. Even severe dislocation is often easy to reposition, decompress and stabilize from the anterior. However, if this does not succeed, a dorsal procedure may be necessary (49). There is no difference in fusion rates, postoperative alignment, neurological outcome or long-term complications between the different approaches. Combined anteroposterior stabilizations prove to be advantageous in biomechanical studies and allow early mobilization of patients (48). This should initially be taken into account in the case of longer-distance restorations. Stabilization and fusion of anterior or posterior is unavoidable in the treatment of spinal cord injuries in the cervical spine. To maintain the functionality of the cervical spine, short-range fusions and instrumentation should always be performed.

#### 4.3.2 Optimal surgical methods for thoracic and lumbar SCI

The initial surgical therapy of thoracolumbar spinal cord injuries includes surgical reduction with stabilization (51). Even without a laminectomy, complete decompression of the spinal canal can often be achieved. Dorsal decompression in the sense of a laminectomy can also be performed in the case of injuries to the dura, to relieve an epidural hematoma, to further relieve and/or to retrieve/reduce individual bone fragments.

The choice for spinal cord injuries caused by flexion and distraction injury as well as extension and distraction injuries in the thoracolumbar region is reduction with long-distance dorsal stabilization or fusion (49).

An initial ventral procedure for spinal cord injuries of the thoracic spine is not recommended. Biomechanical studies confirmed a significantly more effective immobilization of the fracture in long-range dorsal instrumentation over at least 2 segments cranial and 2 segments caudal of the fracture compared to short-range instrumentation. In the case of a decision for short-range dorsal instrumentation, the use of index screws in the fractured vertebral body increases stability, but overall, the short-range dorsal instrumentation is inferior to the long-range instrumentation. The use of cross connectors also improves rotational stability (52). In addition to the less effective immobilization of the fractures, short-term stabilization leads to an increased occurrence of subsequent degeneration in the sense of junctural kyphosis compared to long-distance stabilization (53). There is no functional disadvantage for the patients due to the long-distance dorsal instrumentation (54, 55). Patients with a spinal cord injury have unstable fractures, so posterior stabilization and instrumentation alone are not always sufficient. Concomitant decompression in the sense of laminectomy should be performed dorsally in order to perform reduction without iatrogenic worsening of neurological symptoms, and to follow up with posterior fusion using long-distance dorsal spondylodesis at the same time (56). In the case of spinal cord injuries, an exclusively anterior approach can be made caudally from the level of BWK 5. In addition to the protection of the autochthonous back muscles with direct anterior compression, anterior decompression proved to be advantages. In addition, biomechanical studies have shown effective stabilization. Overall, isolated anterior restoration in the thoracolumbar area has practically not been able to establish itself, as the possibilities of reduction are significantly limited compared to posterior restoration and a large number of equipment is required, which makes care in emergency situations more difficult (56). The incidence of postthoracotomy syndrome in these cases is up to 31% (57). In summary, the initial ventral procedure is clearly not recommended for spinal cord injuries of the thoracic spine.

If SCI occurs at the thoracolumbar junction without persistent compression of the spinal cord, stabilization with reduction and fusion without accompanying decompression is primarily recommended (49). If there are concomitant disc injuries or even a rupture/split fracture with a high tendency to pseudarthrosis formation, additive anterior treatment is indicated (58). A corporectomy or partial corporectomy with vertebral body replacement and accompanying cancellous saplasty or expandable cages are often the therapy of choice. Surgical decompression of the myelon by reduction in the context of primary surgical care plays an important role in high thoracic spine, while spinal canal narrowing of up to 50% is not relevant in the area of the thoracolumbar junction. In the area of the lower lumbar spine (lumbar spine), traumatic spinal stenosis can remain without neurological consequences up to 80% of cases. However, fractures of the caudal lumbar spine do not lead to a spinal cord injury (59). Injuries of the thoracolumbar junction with intervertebral disc involvement or bursting fractures often require concomitant anterior care. Long-distance instrumentation in the spinal column achieves a high stability of the spinal cord injuries; there is no difference in functionality in the clinical outcomes of SCI patients compared to short-range care.

In summary, CSCI with disc protrusions requires an anterior approach with decompression and single-stage fusion using a cage. Coarse dislocations can often also be adequately positioned and stabilized by ventrally. In order to maintain functionality, short-distance fusions of the cervical spine should be taken into consideration. Early laminectomy, surgical reduction, and long-term stabilization should be performed for thoracic spinal cord injuries to avoid follow-up degeneration.

### 4.4 The impact of surgery-related factors on the post-operation complications of SCI

As for surgery-associated factors, a previous study indicated multi-level operation, increased number of surgical segments, prolonged operational duration, excessive intraoperative blood loss, and increased postoperative drainage time were independent predictors for postoperative wound infection (60). Multi-level operation for patients with CSCI may increase the operational duration, and prolonged operation can increase the risk of operational field pollution, and extend operation duration of the CSCI patients’ bed rest (61). Furthermore, a previous also showed number of surgical segments and operational duration were independent predictors of postoperative pulmonary infection, which was consistent with our results (62). Multi-segmental cervical spinal operations may prolong the operational duration, and an extended operation duration may increase intraoperative blood loss, eventually increasing the risk of postoperative pulmonary complications.

For the treatment of CSCI, steroid pulse has been recommended for facilitating the neurological function recovery, suppressing inflammatory responses, and improving the clinical outcomes for CSCI patients. Nevertheless, it remained to be clarified whether this therapeutic option can achieve the ideal effects. Clinicians have identified that the occurrence of some perioperative complications, including electrolyte disturbances, gastrointestinal bleeding, hyperglycemia, hypertension, and pulmonary infection, significantly increases with the preoperative use of high-dose hormonal treatment. Preoperative use of steroid pulse might facilitate the motor functional recovery in CSCI patients, whereas no significant difference was identified in long-term clinical outcomes (63). Further, other studies showed steroid pulse not only failed to facilitate neurological functional recovery for CSCI patients, but was more likely to result in pulmonary infection (64, 65).

The preoperative laboratory examination (blood routine test, renal function, liver function, serum electrolytes, D-dimer, and fibrinogen), a fundamental and widely used examination, has been recommended as a critical adjunct for evaluation of CSCI patients. The combination of laboratory examination parameters and clinical features can make the risk prediction model of SCI patients more reliable and accurate (66, 67). Integrating peripheral blood cells and inflammatory biomarkers may better reflect the complexity of an infection than one single factor, which can contribute to a more reliable prediction of a beginning but not yet clinically apparent pulmonary or wound infection (68). Key molecules and cells can help to identify post-operation complications before clinical or paraclinical signs prompt further diagnostic work-up leading to the diagnosis of the complications after surgical decompression.

### 4.5 Surgical decompression in animal models

Animal models have contributed to a better understanding of the pathophysiology in SCI and have been widely used in the preclinical testing of new therapeutic strategies. Previous study based on animal experiments showed spinal cord ischemia resulted from hemorrhage and swelling may reach peaks at 8 h post SCI (69). The ideal animal models should anatomically and physiologically resemble SCI in human, require minimal training, be inexpensive and can provide consistent results. Rat models are the most frequently utilized for the research of SCI and are well established and inexpensive, and the injury responses are similar to that identified in human SCI, such as the generation of cystic cavities, formation of glial scar and alterations in the extracellular matrix (ECM) (2). Nevertheless, differences exist in anatomy, size, signaling pathways, and the recovery ability after SCI, which have made the direct translation extremely challenging (70). Unfortunately, various treatments in SCI have been unsatisfactory when translated to homo sapiens from small animal models, because of the inherent biological differences. However, larger animal models, like non-human primates, may overcome these barriers partially, whereas substantial differences in costs and strict housing requirements have made their use less frequent (71). Nevertheless, large animal models may establish critical intermediary models to validate results from rodents through producing data of high safety, biodistribution, and feasibility (72). Evaluating novel surgical strategies in various species is a significant way to bolster pre-clinical evidence before performing clinical trials.

Various pre-clinical studies utilizing different SCI animal models showed the benefits of early spinal cord decompression. A previous study showed that monkeys with 1-min spinal cord compression exhibited better electrophysiological recovery and decreased adverse effects on spinal cord blood flow, compared to animals that underwent spinal cord compression for more than 3 min (73). Further, Rabinowitz et al. (74) performed a prospective and randomized study in dogs comparing early surgical decompression (6 h) with or without methylprednisolone as compared with methylprednisolone alone. It showed that surgical decompression with or without methylprednisolone administration offered better neurological improvement than the use of methylprednisolone alone.

### 4.6 Molecular and cellular mechanisms of surgical decompression in SCI

The molecular and cellular mechanisms of neural regeneration after surgical decompression in SCI remain fragmentary. Dhillon et al. (75) showed that axonal plasticity was identified during the recovery process of injured spinal cord after decompressive surgery in a rat model of cervical spondylotic myelopathy (CSM). Further, decompressive surgery can attenuate expression of amyloid precursor protein (APP) and increase expression of growth associated protein 43 (GAP43) (75). The accumulation of APP in nerves indicates that there is a certain degree of cytoskeletal breakdown in injured spinal cord (75). GAP43 plays an important role in the axonal regeneration and budding, which is a known biomarker of axonal regeneration in neuronal cells (76). A previous study reported that surgical decompression may decrease the expression of Cacna2d2 (α2δ2) in the injured spinal cord of CSM rat models, and α2δ2 may modulate the expression of GAP43 in the axonal repair of injured spinal cord after surgical decompression (77).

A recent study assessed 5-hydroxytryptamine (5HT) immunohistochemistry after surgical decompression of SCI, which showed a significant increase of serotonergic fibers, below, above, and within the lesion of injured spinal cord (75). This increase might be regulated by localized sprouting responses of serotonergic fibers. The increase of serotonergic fibers is caused by regeneration of axon, which can be reflected by the concomitant presence of GAP43 detected below, above, and within the lesion of previous compression (78, 79). Additionally, after surgical decompression, re-expression of synaptophysin was identified. Expression of synaptophysin may represent the functional synapses (80, 81). These findings demonstrated that surgical decompression may trigger regenerative responses in axons that can help to establish new functional connections after SCI, which required further investigation.

A recent study identified marked microglial responses to surgical compression in SCI rat models which extended to the adjacent tissues below and above the lesion site (75). After surgical decompression, activation of microglia was significantly reduced, which can reduce the inflammatory responses in SCI. Microglia are the resident immune cells and core sensors of danger signals in the spinal cord. After stimulation, resident M2 type (anti-inflammatory) microglia may polarize to the M1 type (pro-inflammatory) (82), which can secrete pro-inflammatory factors (83). Inflammasomes activation in microglia may induce metabolic shift from oxidative phosphorylation to glycolysis, which can contribute to the polarization of microglia to M1 type, eventually leading to neuroinflammation and neurodegeneration (84). Further, a previous study showed that astrocytes were unable to recover after surgical decompression (75). It is suggested that the decreased activity of astrocytes might have attenuated demyelination and promoted axonal sprouting, as identified after experimental regulation of astrocytes in experimental SCI models (85). Unfortunately, there are very few studies evaluating the alterations of microglia and astrocytes in human SCI and SCI animal models (86).

### 4.7 Applications of artificial intelligence-based technologies combined with surgical decompression in SCI

**Figure 10:**
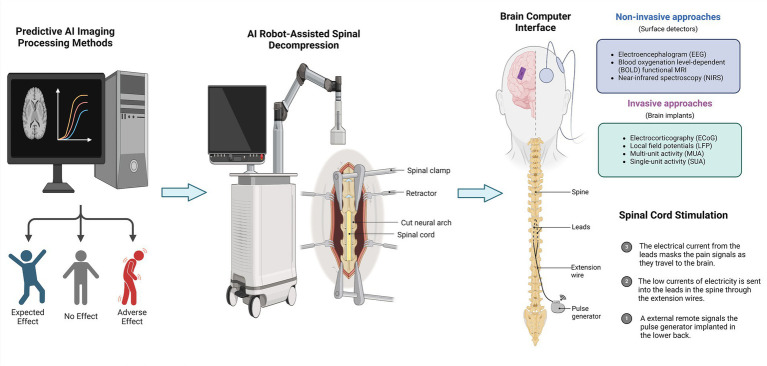
Diagram of hotspots of artificial intelligence (AI) research in spinal cord injury and surgical decompression. AI technology has made breakthroughs in spinal cord neural injury and restoration in recent years. We pinpointed several research hotspots for AI research in spinal cord neural injury and surgical decompression: (1) predictive AI imaging processing methods; (2) AI robot-assisted spinal decompression; (3) AI-based brain computer interface; (4) and AI-assisted spinal cord stimulation, positively promoting functional recovery.

Recently, various breakthroughs have been made for the applications of artificial intelligence (AI) in SCI and rehabilitation, such as predictive AI imaging processing methods, AI robot-assisted spinal decompression, AI-based brain computer interface, and AI-assisted spinal cord stimulation, positively promoting functional recovery (Figure 10). AI has been widely applied in neural imaging for patients with SCI. Convolutional neural network (CNN)-based image segmentation models could make tremendous contributions to imaging parameters, accurate diagnosis, and risk stratification of SCI patients (87). AI can be used to track and investigate all neural components of injured spinal cord in real-time, such as neural structure and neuroplasticity (88). Intelligent robots and limb exoskeletons for assisted surgery and rehabilitation emerged as an important research hotspot in the field of SCI and surgical decompression (89). Fang et al. emphasized that robot-assisted gait training (RAGT) can improve the spasticity syndromes and walking capability in patients with SCI, which can normalize muscle tone and improve lower extremity function in patients with SCI without resulting in extra pain (90). AI-based rehabilitation robots are interactive motorized devices which can realize precise measurements and fine limb movements (91). In the future, more efforts can be devoted to developing novel AI models, including supervised learning and feed-forward topological neural networks (92, 93), to enhance the tolerance, safety, and walking functional efficacy of robotic exoskeletons to promote the functional recovery of SCI patients. Furthermore, applications of AI have also been used to repair SCI based on nano-biomaterial technology (94). Sten cell transplantation in the lesions of SCI is a promising therapeutic strategy. However, it remained challenges due to the inflammatory microenvironment within the lesions (95). Using AI for fabricating polymeric nanomaterials could provide the microenvironment needed for the repair and regrowth of neural stem cells (NSCs) to promote remodeling and repair of neural cells (96). Specifically, Li et al. (94) synthesized a 3D-bioactive scaffold and showed that neural networks derived from NSCs modified by pro-myosin receptor kinase C exhibited excellent viability in the scaffold. Moreover, Yuan et al. (97) synthesized DNA hydrogel with high permeability properties using AI to repair a 2-mm spinal cord gap in SCI rat models and implanted the proliferation and differentiation of endogenous stem cells for establishing a nascent neural network. They showed that neural network organization established through transplantation in 3D-bioactive scaffolds was a candidate therapeutic strategy for SCI. Nevertheless, this AI-based nanotechnology has not yet been investigated on a broader scale, and researchers can focus on this research topic in the near future.

Brain-computer interfaces with deep learning algorithms are also one of the latest hotspots in this research field. Brain-computer interface devices are be used to restore lost function and construct electronic “neural bypasses” for circumventing damaged pathways in the injured spinal cord (98, 99). AI techniques applied to brain-computer interfaces can enable disabled patients to control machines as well as other devices. Using implanted intracortical brain-computer interfaces, the patient’s cortical signals could be utilized to direct limb movements (100). For instance, Collinger et al. (101) implanted two 96-channel intracortical microelectrodes in a patient’s motor cortex and assessed that quadriplegic patients could use the brain-computer interface to realize neural control of high-performance prostheses quickly. Additionally, Ajiboye et al. (101) restored limb movement of paralyzed patients via implanting intracortical brain-computer interfaces and functional electrical stimulation components. They suggested that neuro-electrical stimulation and intracortical brain-computer interface techniques can be integrated to improve the motor and neurophysiologic status of patients with SCI more effectively. In the future, machine learning algorithms can be used to regulate the activation of nerves and muscles in patients with SCI based on high-resolution and customized electrical stimulation devices. Future neuro-electrical stimulation research could combine AI and deep learning methods like CNNs, and utilize multiple strategies to modulate the neurological and physiological status and promote motor functional recovery in patients with SCI after surgery. Importantly, developing more targeted neuro-electrical and spinal cord stimulation techniques through a wide range of spatially selective stimulation strategies may be critical future research directions.

It is important to emphasize that the data retrieval process in our work may result in sampling bias to some extent. Firstly, the exclusive reliance on literature in English may cause neglect of valuable perspectives from non-English documents. Secondly, the informative feature of bibliometric analysis may potentially overlook unique and niche aspects of the research field. Therefore, when extrapolating our findings to the wider population, caution should be exercised.

## 5 Conclusion

This study provided a comprehensive outlook of the research on SCI and surgical decompression spanning the last 5 decades. The publication number has significantly increased since 2005, and there is a potential of further growth in scientific output in the near future. Increasing multidisciplinary collaborations and thematic evolutions were identified in this field, revealing the significant role of surgical decompression in SCI therapeutic outcomes. The USA, China, and Japan were the most productive countries in this field, while collaboration among countries in this field should be further promoted. *Spine*, *Spinal Cord*, and *Journal of Neurosurgery* were the most impactive journals. Michael G. Fehlings and Alexander R Vaccaro were the most productive authors. The University of Toronto was the leading affiliation with the highest publication number. While the recommendations for early surgical decompression in specific cases of acute SCI are well recognized, there is considerable uncertainty regarding the role of and the timing of surgical decompression of the spinal cord in the management of SCI patients. Most of the studies defined early surgical decompression as decompression in 24 h post SCI, which was related to better functional recovery in patients with acute SCI. Early surgical decompression exhibited better neurological outcomes in subpopulations according to the injury degree and whether or not preoperative steroid pulse were utilized. Beneficial effects on surgical decompression in 8 h post SCI has been discussed recently, whereas further studies are still urgently needed. Surgical decompression can inhibit microglia activation and trigger regenerative responses in axons that can help to establish new functional connections after SCI, while the underlying molecular and cellular mechanisms, as well as potential signaling pathways required further investigation. AI systems and research on spinal surgical decompression and rehabilitation can mutually reinforce each other and drive medical innovation in the near future.

## Data availability statement

The original contributions presented in the study are included in the article/Supplementary material, further inquiries can be directed to the corresponding authors.

## Author contributions

SW: Conceptualization, Data curation, Formal analysis, Funding acquisition, Investigation, Methodology, Project administration, Resources, Software, Supervision, Validation, Visualization, Writing – original draft, Writing – review & editing. WX: Conceptualization, Data curation, Investigation, Methodology, Software, Supervision, Writing – original draft. JW: Conceptualization, Data curation, Formal analysis, Investigation, Resources, Supervision, Writing – original draft. XH: Writing – review & editing, Conceptualization, Data curation, Formal analysis, Investigation, Methodology, Software, Supervision. ZW: Writing – review & editing, Conceptualization, Data curation, Formal analysis, Investigation, Methodology, Resources, Software, Supervision, Validation, Visualization. CL: Data curation, Investigation, Methodology, Resources, Software, Supervision, Validation, Writing – review & editing. ZX: Conceptualization, Data curation, Formal analysis, Investigation, Methodology, Software, Supervision, Validation, Writing – review & editing. BM: Data curation, Formal analysis, Investigation, Methodology, Software, Supervision, Writing – review & editing, Conceptualization. LC: Conceptualization, Data curation, Formal analysis, Funding acquisition, Investigation, Methodology, Project administration, Resources, Software, Supervision, Validation, Visualization, Writing – original draft, Writing – review & editing.

## Supplementary material

SUPPLEMENTARY FIGURE S1 The Data retrieval strategies and collection process of this study.

SUPPLEMENTARY FIGURE S2 Analysis of core sources in the field of spinal cord injury (SCI) and surgical decompression. **(A)** The top 10 most productive journal in SCI and surgical decompression researches. **(B)** Core journals of SCI and surgical decompression researches based on Bradford’s Law.

SUPPLEMENTARY FIGURE S3 Supplementary materials for author analysis in the field of spinal cord injury (SCI) and surgical decompression. **(A)** The frequency distribution of scientific productivity in SCI and surgical decompression researches by Lotka’s law. **(B)** Top 10 most local cited authors in SCI and surgical decompression researches. **(C)** Top 10 local impactive authors measured by H-index in SCI and surgical decompression researches.

SUPPLEMENTARY FIGURE S4 Supplementary materials for keyword analysis in the field of spinal cord injury (SCI) and surgical decompression. **(A)** Word Cloud of the top 50 frequent keywords in SCI and surgical decompression researches. **(B)** Tree Map of the top 50 frequent keywords in SCI and surgical decompression researches.
