# Supplementary figures and images for: Tracing the evolving dynamics and research hotspots of spinal cord injury and surgical decompression from 1975 to 2024: a bibliometric analysis
Source: Front Neurol. 2024 Aug 5;15:1442145. doi: 10.3389/fneur.2024.1442145 (PMC11330800; doi:10.3389/fneur.2024.1442145)

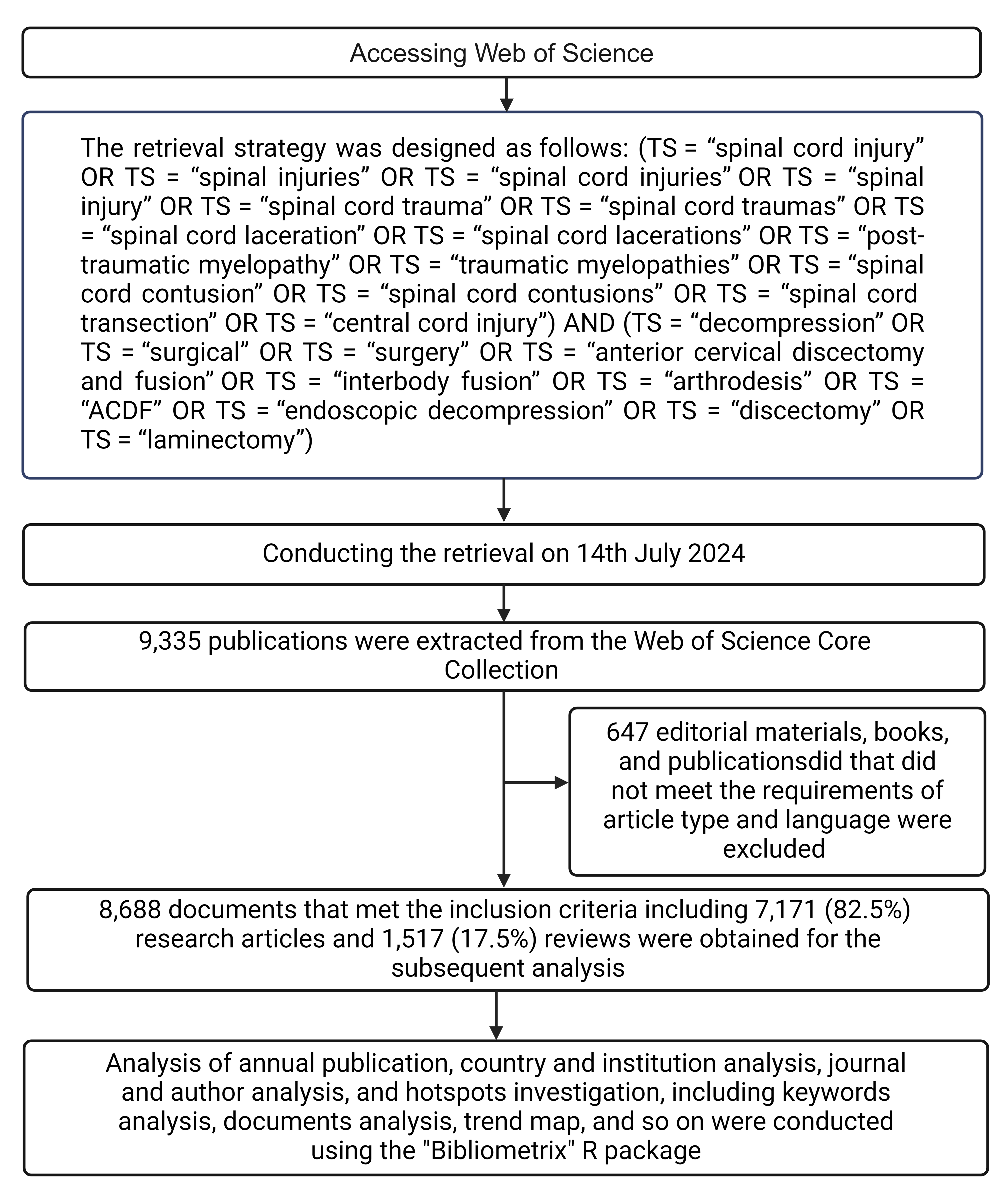

Supplement: SUPPLEMENTARY FIGURE S1 — The Data retrieval strategies and collection process of this study. [file Image_1.TIF]

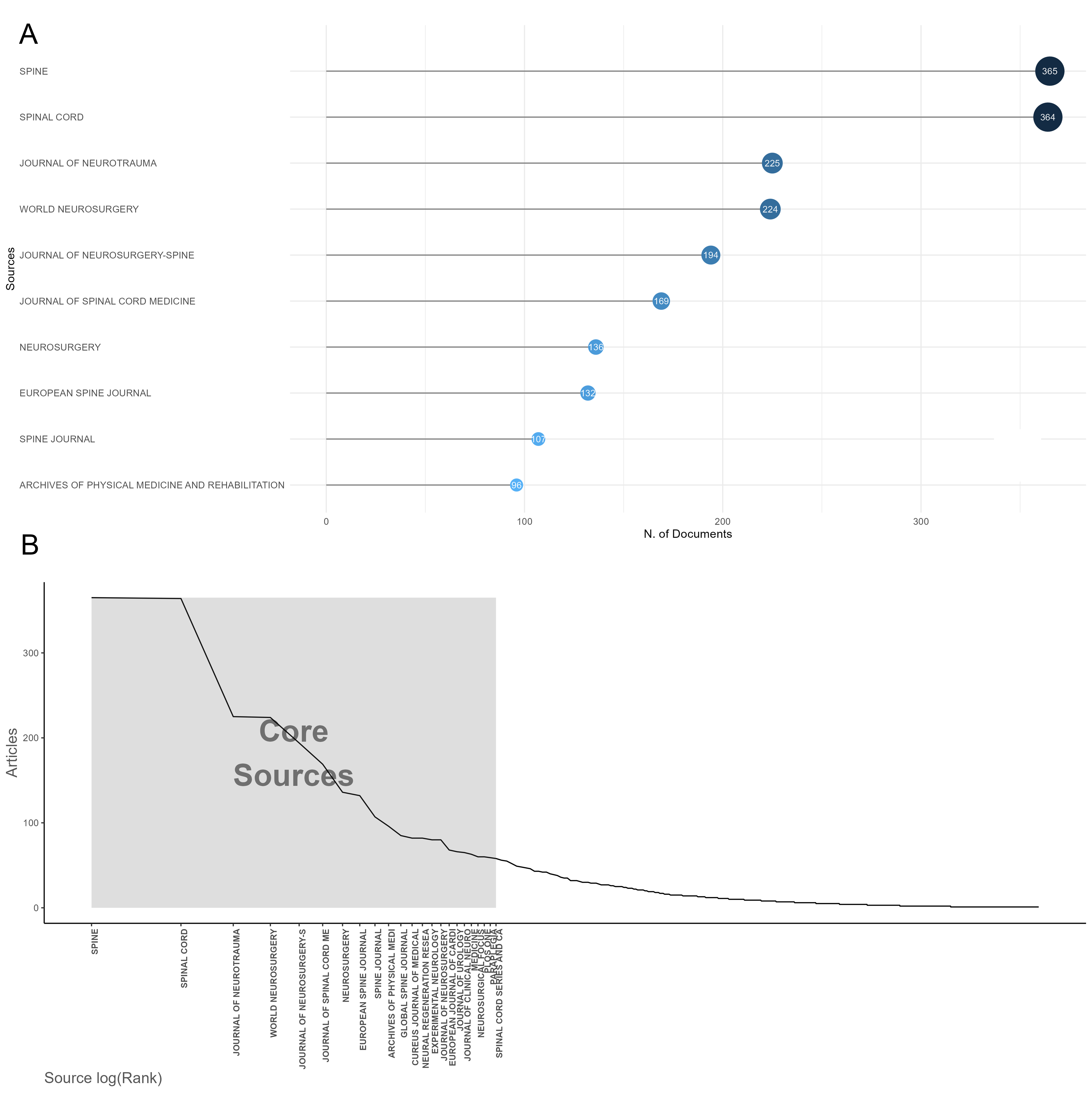

Supplement: SUPPLEMENTARY FIGURE S2 — Analysis of core sources in the field of spinal cord injury (SCI) and surgical decompression. (A) The top 10 most productive journal in SCI and surgical decompression researches. (B) Core journals of SCI and surgical decompression researches based on Bradford’s Law. [file Image_2.TIF]

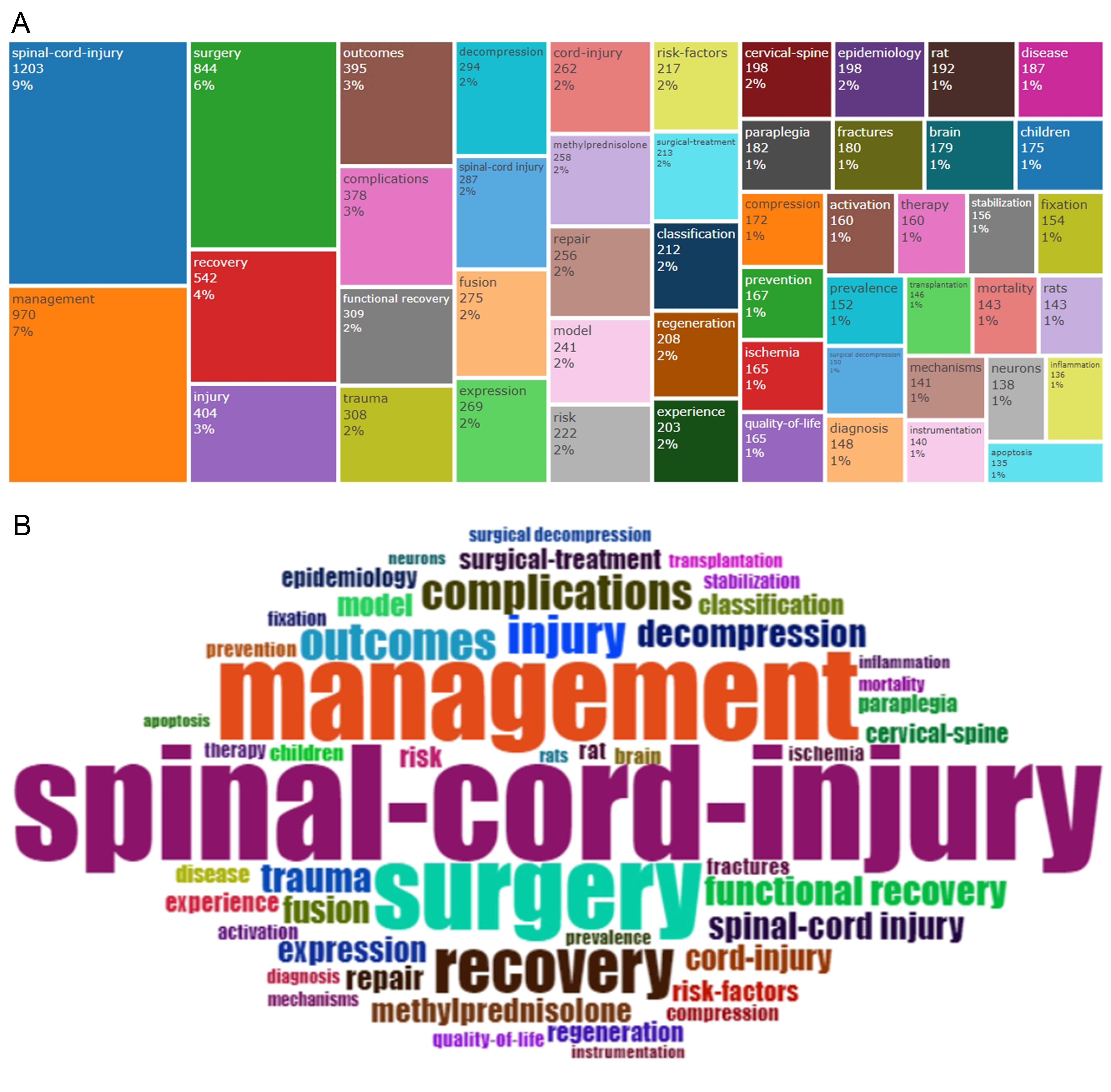

Supplement: SUPPLEMENTARY FIGURE S4 — Supplementary materials for keyword analysis in the field of spinal cord injury (SCI) and surgical decompression. (A) Word Cloud of the top 50 frequent keywords in SCI and surgical decompression researches. (B) Tree Map of the top 50 frequent keywords in SCI and surgical decompression researches. [file Image_4.TIF]
